# Parallelized Particle Swarm Optimization on FPGA for Realtime Ballistic Target Tracking

**DOI:** 10.3390/s23208456

**Published:** 2023-10-13

**Authors:** Juhyeon Park, Heoncheol Lee, Hyuck-Hoon Kwon, Yeji Hwang, Wonseok Choi

**Affiliations:** 1 School of Electronic Engineering, Kumoh National Institute of Technology, Gumi 39177, Republic of Korea; 2 Department of IT Convergence Engineering, Kumoh National Institute of Technology, Gumi 39177, Republic of Korea; 3 PGM R&D Lab, LIGNEX1, Seongnam 13488, Republic of Korea

**Keywords:** ballistic target tracking, field-programmable gate array, particle swarm optimization, realtime system

## Abstract

This paper addresses the problem of tracking a high-speed ballistic target in real time. Particle swarm optimization (PSO) can be a solution to overcome the motion of the ballistic target and the nonlinearity of the measurement model. However, in general, particle swarm optimization requires a great deal of computation time, so it is difficult to apply to realtime systems. In this paper, we propose a parallelized particle swarm optimization technique using field-programmable gate array (FPGA) to be accelerated for realtime ballistic target tracking. The realtime performance of the proposed method has been tested and analyzed on a well-known heterogeneous processing system with a field-programmable gate array. The proposed parallelized particle swarm optimization was successfully conducted on the heterogeneous processing system and produced similar tracking results. Also, compared to conventional particle swarm optimization, which is based on the only central processing unit, the computation time is significantly reduced by up to 3.89×.

## 1. Introduction

The performance of target tracking with ballistic trajectories and interception can be determined by the accuracy of target tracking. Therefore, in order to track a target, it is necessary to select an algorithm that can accurately estimate the state of the target’s location, angle, etc. While model noise from measuring the state of a target is typically assumed to have a Gaussian distribution for mathematical simplicity, the measurement model noise generated by radome seekers and scintillators is nonlinear and non-Gaussian in nature [1,2], so assuming a Gaussian distribution is not appropriate. Some filtering-based algorithms do not show satisfactory performance in accurately tracking targets due to uncertainties in nonlinear and non-Gaussian properties. Linear Kalman-filter-based target tracking algorithms also have a problem in that values do not converge or diverge while estimating the state of a target.

Various nonlinear filters such as extended Kalman filter (EKF), particle filter (PF), and unscented Kalman filter (UKF) were applied to estimate the state of the target to solve problems caused by the nonlinear and non-Gaussian properties of noise [3,4,5]. Optimization methods can also be applied to estimate the state of the target in environments with nonlinear and non-Gaussian noise. Among them, particle swarm optimization (PSO) methods are being actively studied by applying them to estimating the state of the target because they can handle various error distributions. Also, due to the characteristics of PSO methods, the particles exchange information with each other to find the optimal point, so even if some particles do not find the exact value and do not converge, the global optimum is eventually reached. However, the main limitation of using particle swarm optimization methods is that, to find the optimal value, the number of particles and the number of epochs, meaning the number of times the particles move, must be large, and the performance is proportional. Therefore, applying PSO to realtime systems has the limitation of reducing the computational time of finding an appropriate compromise between performance and realtime properties.

In this paper, a particle swarm optimization method capable of estimating the state of the target in a nonlinear and non-Gaussian noise environment is used for precise realtime tracking and intercepting ballistic targets. However, a large number of particles and a large number of epochs are required to accurately estimate the state of the target using the PSO method. The PSO method is a sampling-based algorithm, so, as the number of particles used and the number of epochs increase, the time required increases. Therefore, acceleration is essential in order for the PSO method to have real time. In our previous work, we accelerated the ballistic target tracking algorithm using the design of heterogeneous devices of a central processing unit (CPU) and a graphics processing unit (GPU) in an on-board environment [6]. As a result, parallelization using GPU could benefit greatly in terms of the time of the algorithm. Parallel acceleration using GPU has the advantage of being able to conduct parallel research on a relatively convenient platform through the CUDA Library. However, acceleration using GPUs consumes a great deal of power and generates a great deal of heat when performing parallel operations on thousands of internal cores. For the Jetson Xavier NX used in previous studies, it basically consumes 15 W of power and consumes more or less depending on the nvpmodel. The heat generation problem caused by high power consumption cannot be ignored. In the case of Xavier NX, considerable heat was generated even though it contained a heat sink. These power consumption and heat generation problems are quite sensitive in the defense sector, and it is necessary to reduce power consumption and heat generation for the stability of the system. The design of heterogeneous devices of FPGA and CPU can overcome these power consumption and heat generation problems. FPGA has disadvantages in terms of price compared to GPU, but it has the advantage of low power consumption and low heat generation because it is completed within a single core designed through software. Due to these advantages, algorithm acceleration using FPGA is being carried out in various fields, as shown in Table 1. In this paper, a part that takes a great deal of calculation time during PSO was identified, and then parallelization using FPGA was performed on the part. The acceleration was carried out using the AMD Zynq 7000 SoC ZC706 Evaluation Kit equipped with both a processing system (PS) and programmable logic (PL) to suit the onboard environment under the assumption that state estimation is carried out using PSO methods in ballistic target interceptors. As a result, PSO in an embedded environment was mutually designed with PS and PL to improve real time and successfully estimate the state of the ballistic target.

## 2. Background

The PSO method is a metaheuristic optimization algorithm that imitates the natural phenomenon of individuals using their collective intelligence to find the optimal solution [7], and its performance and utility have been proven over a long period of time [8,9,10,11]. PSO methods are being researched to solve problems in a variety of fields, including construction, edge computing, and energy [12,13,14]. Especially, the PSO method has the advantage of being simple and easy to implement compared to other metaheuristic optimization algorithms, and its ability to handle various types of error distributions makes it applicable to state estimation of goals. Table 1 shows papers related to PSO methods for target estimation. There is a great deal of research going on [15,16,17], and relevant research for missile applications can be found [18,19,20]. Also, various attempts have been made to accelerate the algorithm to overcome the limitations of the PSO method described in Section 1. First, acceleration using GPUs has been researched [21,22,23]. In addition, as in this paper, acceleration studies using FPGA can be found [24,25,26,27].

Previous acceleration research has been completed in various sections, depending on the characteristics of the application. In this paper, a PSO method is applied to estimate ballistic targets, and there are various parts with a large proportion of computational time. Accordingly, many parts with large computational time are modularized and parallelized, respectively. This modularization allows the application to operate more flexibly and adaptively. Therefore, unlike other studies, this paper has various sections to be parallelized, and, accordingly, parallel acceleration was performed for all parts of random value to particles, predicted measurements, associated likelihood function, and update particles.

The contributions of this paper are as follows.

- To the best of our knowledge, this is the first approach to accelerate PSO for ballistic target tracking with an FPGA.
- This paper has parallelized most of the computationally time-consuming parts of the PSO.
- A new parallelization method for realtime ballistic target tracking has been developed.
- The proposed approach has been validated on a real embedded system, and the computation time has been significantly reduced.

The rest of this paper is organized as follows: Section 3 describes the missile target tracking system based on particle swarm optimization and the realtime problem with particle swarm optimization. In Section 4, after profiling the computational time for the entire particle swarm optimization, a new parallelization method is proposed for the computational-intensive parts. Section 5 provides a description of the experimental environment, a performance evaluation of each parallelization part, and a performance evaluation of the overall algorithm. Finally, Section 6 presents our conclusions.

## 3. Problem Description

The end goal of the algorithm for realtime ballistic target tracking is to estimate the state of the target in real time. The algorithm is experimented in a simulation environment to evaluate the performance of the algorithm. Therefore, it is essential to simulate the trajectory of the ballistic missile target in the simulation environment. Aerodynamic forces such as gravity and drag have a major impact on the path of a ballistic missile in the atmosphere, unlike outside the atmosphere. In this paper, we focus on gravitational and aerodynamic forces because our goal is to estimate the target state of the missile after it has reentered the atmosphere. As a result, we simulate the situation by assuming that the missile is a point mass in a three-dimensional Cartesian coordinate system.

The three-dimensional nonlinear motion of the simulated missile, including gravitational and aerodynamic forces, can be modeled as follows [28].

(1) $$\dot{pos\_x}=Vcos\gamma cos\psi \;\;\;\;\;\;\;\dot{pos\_y}=Vcos\gamma sin\psi \;\;\;\;\;\;\;\dot{pos\_z}=-Vsin\gamma$$

(2) $$\dot{V}=\frac{T-D-mgsin\gamma }{m}\;\;\;\;\;\;\;\dot{\gamma }=\frac{Lcos\delta -mgcos\gamma }{Vm}\;\;\;\;\;\;\;\dot{\psi }=\frac{Lsin\delta }{Vmcos\gamma }$$

(3) $$D=\frac{1}{2}\rho V^{2}\times E_{D}\times S\;\;\;\;\;\;\;L=\frac{1}{2}\rho V^{2}\times E_{L}\times S$$

where pos_x, pos_y, and pos_z represent the position of the missile, and V represents the velocity, and γ and ψ represent the altitude and azimuth, respectively. Also, m is the mass, g is the gravitational constant, and T, D, and L are thrust, drag, and lift, respectively. Aerodynamic forces are composed of air density ρ, drag coefficient $E_{D}$, lift coefficient $E_{L}$, and, finally, the reference area S. δ represents the direction of lift generation. As mentioned earlier, the goal of this approach is to estimate the state of the missile during the reentry phase. In general, during the reentry phase, the T is set to zero and m is assumed to be constant because the propellant of the missile has finished burning. Also, within the atmosphere, the L is assumed to be zero because the ballistic missile’s maneuvers are generally very small and have a small effect compared to drag.

### 3.1. The Problem of Target Tracking

In this paper, target tracking is based on the well-known Singer motion model [29,30]. The Singer motion model assumes that the target is a first-order static Markov process with zero mean. The state space representation of the Singer model in continuous time is defined as follows.

(4) $$\dot{x}=Sx+Yw$$

(5) $$S=\left[\begin{array}{ccc} 0_{3} & J_{3} & 0_{3}\\ 0_{3} & 0_{3} & J_{3}\\ 0_{3} & 0_{3} & -\frac{J_{3}}{\tau } \end{array}\right],\;Y=\left[\begin{array}{c} 0_{3}\\ 0_{3}\\ J_{3} \end{array}\right]$$

where $\dot{x}$ is the state of the tracked target, w is white Gaussian noise with mean zero and time constant τ. $J_{3}$ of the S and Y matrices is a cubic identity matrix, and τ is the mobilization constant.

The discrete time equation for white Gaussian noise w is defined as follows.

(6) $$x_{i}=\Omega_{i-1}x_{i-1}+w_{i-1},\;\;\;\;\;\;\;w_{i-1}\sim N\left(0,R_{i}\right)$$

(7) $$\Omega_{i}≅J+S∆t$$

(8) $$R_{i}≅H_{w}R_{0}=H_{w}\left[\begin{array}{ccc} \frac{∆t^{5}}{20}J_{3} & \frac{∆t^{4}}{8}J_{3} & \frac{∆t^{3}}{6}J_{3}\\ \frac{∆t^{4}}{8}J_{3} & \frac{∆t^{3}}{3}J_{3} & \frac{∆t^{2}}{2}J_{3}\\ \frac{∆t^{3}}{6}J_{3} & \frac{∆t^{2}}{2}J_{3} & ∆tJ_{3} \end{array}\right]$$

where $\Omega_{i}$ denotes the state transition matrix and ∆t denotes the sampling interval. The covariance $R_{i}$ is composed of $H_{w}$, the power spectral density and $R_{0}$, the white noise jerk model. The magnitude of the increase in acceleration over a period of time is represented by the jerk integral over that time.

The state variable x is defined as follows.

(9) $$x={\left[P^{T}V^{T}A^{T}\right]}^{T}$$

(10) $$P={\left[xyz\right]}^{T}$$

where *P*, *V*, and *A* are position, velocity, and acceleration in the Cartesian coordinate system, respectively, and [xyz] is the position of the target in the three-dimensional Cartesian coordinate system.

With this definition of the target, next define the data being measured. First, assume that the measurements of the target are made by radome seeker, which measures altitude, attitude, and distance. These measurements can vary depending on the relative position of the target and the radar, defined as follows.

(11) $${\left[x_{a}y_{a}z_{a}\right]}^{T}={\left[xyz\right]}^{T}-{\left[x_{s}y_{s}z_{s}\right]}^{T}$$

where $x_{a}$, $y_{a}$, and $z_{a}$ represent the relative positions of the target and the radar, and $x_{s}$, $y_{s}$, and $z_{s}$ represent the radar positions. Therefore, the two bearing angles and the relative distance can be expressed as follows.

(12) $$\left[\begin{array}{c} c_{D}\\ c_{\theta }\\ c_{\psi } \end{array}\right]=\left[\begin{array}{c} \sqrt{x_{a}{}^{2}+y_{a}{}^{2}+z_{a}{}^{2}}+R\_n_{D}\\ {tan}^{-1}\left(\frac{z_{a}}{\sqrt{x_{a}{}^{2}+y_{a}{}^{2}}}\right)+G\_n_{\theta }+R\_n_{\theta }\\ {tan}^{-1}\left(\frac{y_{a}}{x_{a}}\right)+G\_n_{\psi }+R\_n_{\psi } \end{array}\right]$$

where $R\_n_{D}$, $R\_n_{\theta }$, $R\_n_{\psi }$ means the radar receiver noise, which is Gaussian noise, and $G\_n_{\theta }$, $G\_n_{\psi }$ means the non-Gaussian glint noise, which can be called the radar measurement error.

### 3.2. The Problem of Real Time

**Figure 1:**
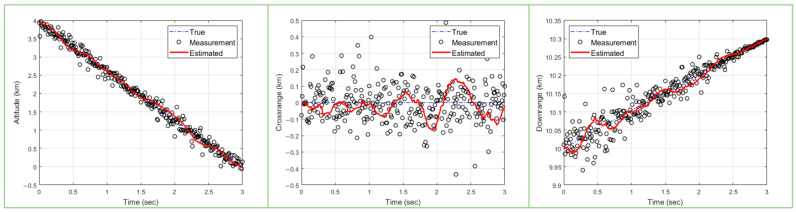
Ballistic target estimation results with 50 particles and 20 epochs (blue dashed line: true/circle: measurements/red solid line: tracking results).

**Figure 2:**
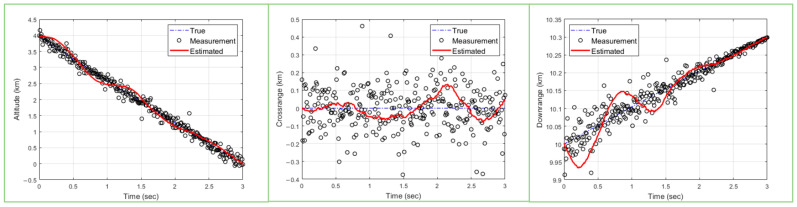
Ballistic target estimation results with 200 particles and 5 epochs (blue dashed line: true/circle: measurements/red solid line: tracking results).

Precision guidance and control to successfully intercept a target are highly dependent on how accurately and quickly the target can be tracked. Therefore, accuracy and fast updates are critical for algorithms that track high-speed targets such as ballistic missiles. In this paper, a PSO algorithm is used for high-speed ballistic target tracking. Due to the characteristics of sampling-based algorithms, PSO methods need to ensure a sufficient number of particles and a sufficient number of epochs to estimate the target with high accuracy. Figure 1 shows the results of ballistic target tracking using PSO with 50 particles and 20 epochs. Next, Figure 2 shows the results of ballistic target tracking using the PSO technique with 200 particles and 5 epochs. In Figure 1 and Figure 2, the left plot shows the estimated altitude range compared to the true model, the middle plot shows the crossrange compared to the true model, and the right plot shows the target downrange compared to the true model. Further, we can see that the red line, which is the result of estimating the state of the target, deviates significantly from the actual state of the target shown by the blue line. In conclusion, both experiments failed to accurately estimate the target’s state. This shows that the PSO algorithm requires a larger number of particles and epochs to estimate the accurate state of the target.

In order to intercept a ballistic trajectory target, it is necessary to be able to estimate the target’s state in real time. However, in the previous experiment, ballistic target estimation using PSO requires a sufficient number of particles and epochs to be ensured. This directly impacts the real time performance of the application. In the case of PSO, as the number of particles and epochs increases, the computation time increases, so there is a trade-off between the accuracy and real time of the target estimation. To overcome this trade-off problem, this paper proposes a parallel acceleration method through heterogeneous device co-design of CPU and FPGA. When the PSO algorithm is performed using only the CPU, the algorithm is performed sequentially, which greatly increases the computation time. However, in FPGA, the same operation can be parallelized and computed simultaneously, which can reduce the computation time compared to the method using only CPU.

## 4. Proposed Method

### 4.1. Overview of the Proposed Method

**Figure 3:**
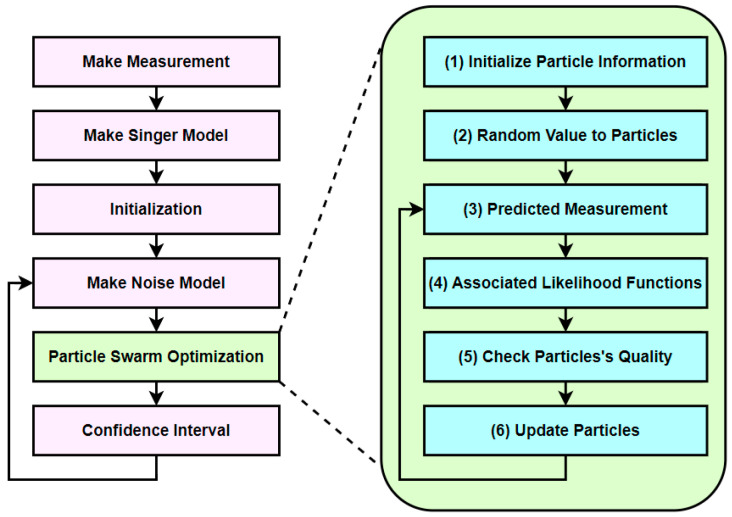
Flowchart of target tracking algorithm.

The overall flow of the application for ballistic target tracking is shown in Figure 3. The PSO algorithm applied to the application is divided into (1) initialize particle information, (2) random value to particles, (3) predicted measurements, (4) associated likelihood functions, (5) check particle’s quality, and (6) update particles. In this paper, the computation time for each part is measured to improve the computation speed of the algorithm, and a method for accelerating the parts that take a long time is proposed.

### 4.2. Computation Time Profiling

**Figure 4:**
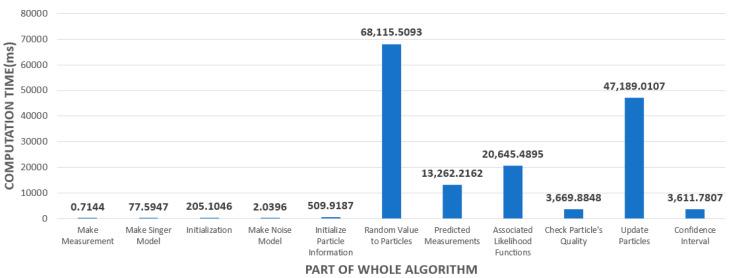
Computation time of the target tracking algorithm using particle swarm optimization.

The computation times for each part were measured with 3000 particles and 10 epochs on the PS of the AMD Zynq 7000 SoC ZC706 Evaluation Kit, and the results are shown in Figure 4. The results of Figure 4 show that (2) random value to particles takes the longest time, followed by (6) update particles, (4) associated likelihood function, and (3) predicted measurement. Since the computational time of these four parts accounts for about 94.87% of the total algorithm, it needs to be accelerated. Therefore, these four sections are set as targets for parallelization and acceleration using the PL of ZC706’s FPGA to enhance performance. The FPGA of the PL was utilized for the four sections, and the PS was used for the remaining sections, resulting in a heterogeneous device design using both the CPU and FPGA.

### 4.3. Parallelization Method #1: Random Value to Particles

The random value to particle process accounts for about 43.31% of the entire algorithm and is the most computationally time-consuming part of the algorithm. Therefore, parallelizing this part is essential in a realtime target tracking environment. This is performed for every iteration of the PSO, as many times as there are particles. The position of the particle can be calculated as follows.

(13) $$Pt_{updated}=\left(\Omega \times Pt_{hat}\right)+\left(\Omega \times \sqrt{R_{i}}\times randnum\right)$$

where Ω is the state transition matrix of the target and $Pt_{hat}$ is the position information of the initial particle. $R_{i}\;$is the covariance matrix for the noise, and, finally, randnum is a random number generated by a normal distribution with mean 0 and standard deviation 1. In our method, we do not generate random numbers in PL for simplicity of design but use PS to generate random numbers according to the Mersenne Twister method and then transfer them to PL [31]. The resulting $Pt_{updated}$ is used to find the optimal location in the particle set.

To parallelize this part, we use Xilinx Vitis HLS to synthesize the hardware IP. Algorithm 1 shows pseudocode for the random value to particle part. This part consists of three nested iterations, each repeating the number of particles, the number of rows, and the number of columns of the target’s state transition matrix. It is important to note that the lowest iteration has a compound operator.
**Algorithm 1:** Random Value to ParticlesInputParticle position array $Pt_{hat}$ and Random number array randnum
OutputUpdated particle position array $Pt_{updated}$
1.**for** *i* = 0: the number of particle2.   **for** *j* = 0: the number of rows of transition matrix 3.          Ω_Ri_rand←04.           Ω_pt←05.   **for** k = 0: the number of columns of transition matrix 6.$\;\;\;\;\;\;\;\;\;\;\;\;\;\;\;\;\;\Omega \_Ri\_rand+=\Omega *\sqrt{R_{i}}*randnum$7.                 Ω_pt $+=\Omega *\;Pt_{hat}$8.   **end**9.  $Pt_{updated}=\Omega \_pt+\Omega \_Ri\_rand$
10.  **end**
11.**end**

Designing parallel computation hardware IP to accelerate the random value to particle part is completed as follows. Algorithm 2 shows pseudocode for a parallelized random value to particle. First, the clock of the hardware IP is set to 10 ns based on experimental results that show no negative slack. Next, since the data transfer from PS to PL and PL to PS uses the AXI Stream interface, we need to convert the data type between the transfers. Since the AXI Stream interface supports uint32 type for data transfer, the data type casting part is also added when configuring the hardware IP. Data type casting converts between uint32 type and float type, and this process takes 1 clock per execution. Since the data type casting process takes only 1 clock to execute, pipelining is not possible. Therefore, this part uses the sequential processing method.
**Algorithm 2:** Parallelized Random Value to ParticlesInputParticle position uint32 array $Pt_{hat}$ Random number uint32 array randnum
OutputUpdated particle position uint32 array $Pt_{updated}$
1.**do in sequential: for** *x* = 0: Particle Dimension2.   uint32_to_float_datatype_casting($Pt_{hat})$
3.**end**4.**do in sequential: for** *y* = 0: Particle number × Particle Dimension5.   uint32_to_float_datatype_casting(randnum)6.**end**7.**do in parallel: for** i = 0: the number of particle8.   **do in parallel: for** *j* = 0: the number of rows of transition matrix9.      
Ω_Ri_rand←0
10.      
Ω_pt←0
11.      **do in sequential: for** k = 0: the number of columns12.         
$\Omega \_Ri\_rand+=\Omega *\sqrt{R_{i}}*randnum$
13.          Ω_pt $+=\Omega *\;Pt_{hat}$14.**end**15.      
$Pt_{updated}=\Omega \_pt+\Omega \_Ri\_rand$
16.   
**end**
17.**end**18.**do in sequential: for** *z* = 0: Particle number × Particle Dimension19.   float_to_uint32_datatype_casting($Pt_{updated})$
20.**end**

**Figure 5:**
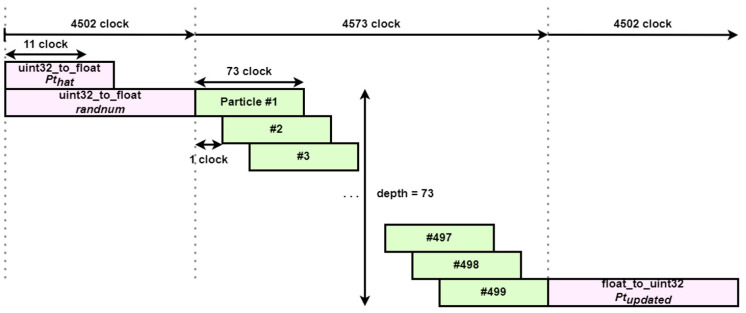
Parallelized random value to particle block diagram.

The main computation part consists of three nested iterations as described above, and the lowest iteration has a compound operator. Compound operands cannot be parallelized because each operation is interdependent. Therefore, the lowest iteration remains sequential, and pipelining is performed for the higher iterations. Parallel operations by pipelining are stacked every 1 clock, and the number of particles input to the hardware IP at a time is set to 500 for flexibility in changing parameters between PS and PL. Therefore, the upper iteration is repeated a total of 4500 times, which is the product of the number of particles and the target’s state transition matrix. The result of this pipelining can be seen in Figure 5. The sequential form of the bottom iteration consumes a total of 73 clocks, which determines the depth of the pipelining to be 73.

### 4.4. Parallelization Method #2: Predicted Measurement and Associated Likelihood Functions

The predicted measurements part of the algorithm, which estimates the state of the target, takes up about 8.43% of the entire algorithm and is the fourth most time-consuming part of the algorithm. This process is performed for an initialized number of epochs in each iteration of the PSO and involves calculating estimates of distance, angle, and rotation angle for each particle. To obtain an estimate of the distance, angle, and rotation of a target in the Cartesian coordinate system, the equation is as follows.

(14) $$Distance=\sqrt{Pt_{p1}{}^{2}+Pt_{p2}{}^{2}+Pt_{p3}{}^{2}}$$

(15) $$\theta =tan^{-1}\left(\frac{Pt_{p3}}{\sqrt{Pt_{p1}{}^{2}+Pt_{p2}{}^{2}}}\right)\times \frac{180}{\pi }$$

(16) $$\psi =tan^{-1}\left(\frac{Pt_{p2}}{Pt_{p1}}\right)\times \frac{180}{\pi }$$

where $Pt_{p1},\;Pt_{p2},\;Pt_{p3}$ are the position information of the particles in the algorithm to find the optimal point in the particle swarm optimization algorithm. For the estimation of the target state, the particle position information in the algorithm is obtained as a matrix with three rows and a column with a size equal to the number of particles used in the particle swarm optimization. Therefore, for an epoch of PSO, the above equations are repeated as many times as the number of particles. Since the equations are repeated as many times as the number of particles and epochs to estimate the target state, the more particles and the more epochs, the more accurate the final state estimate, but the computation time also increases, so parallelization is performed.

The associated likelihood function part, which measures the quality of the particles by putting the estimates from the predicted measurements into a likelihood function to evaluate the quality of the particles, takes up 13.13% of the total algorithm and is the third most time-consuming part of the algorithm. Like the predicted measurements part, this process is performed at each epoch of the PSO and is repeated for the number of particles. As the number of particles increases to improve the accuracy of the estimation, the computation time of this part also increases, so it is necessary to parallelize it. The error values of the estimates of distance, angle, and rotation angle obtained in the previous process can be obtained through their respective likelihood functions. The likelihood function for distance is defined as follows.

(17) $$Distance_{E}=\frac{1}{\sqrt{2\pi }\times sig_{D}}\times exp\left(\frac{-{\left(mea_{D}-Distance\right)}^{2}}{{\left(2\times sig_{D}\right)}^{2}}\right)$$

where $sig_{D}$ and $mea_{D}$ are the measurement noise and the acquired measurement of the target’s distance, respectively, and $sig_{D}$ is set to 1 in this paper. Distance is the estimated distance value obtained in the predicted measurements part.

The likelihood functions and internal operations for angles and rotation angles are defined as follows.

(18) $$sub_{1}=\left(1-ep\right)\times \left(\frac{1}{\sqrt{2\pi \times (sig_{\theta ,\psi }{}^{2}+\left(sig_{1}/Distance^{2}\right))}}\right)$$

(19) $$sub_{2}=exp\left(\frac{-{\left(mea_{\theta ,\psi }-\theta ,\psi \right)}^{2}}{2({\left(sig_{\theta ,\psi }+sig_{1}\right)}^{2}/Distance^{2})}\right)$$

(20) $$sub_{3}=ep\times \frac{1}{\sqrt{2\pi \times (sig_{\theta ,\psi }{}^{2}+\left(sig_{2}/Distance^{2}\right))}}$$

(21) $$sub_{4}=exp\left(\frac{-{\left(mea_{\theta ,\psi }-\theta ,\psi \right)}^{2}}{2({\left(sig_{\theta ,\psi }+sig_{2}\right)}^{2}/Distance^{2})}\right)$$

(22) $$\theta_{E},\;\psi_{E}=sub_{1}\times sub_{2}+sub_{3}\times sub_{4}$$

(23) $$Pt_{E}=Distance_{E}\times \theta_{E}\times \psi_{E}$$

where $sig_{\theta }$ and $sig_{\psi }$ are the measurement noise of angle and rotation angle, respectively, and are set to 0.1, and $sig_{1}$ and $sig_{2}$ are the estimation noise, and are set to 0.5 and 0.1, respectively. ep is the glint probability, which means the probability of noise. Further, $mea_{\theta ,\psi }$ is the measurement obtained for θ and ψ. The likelihood functions for angles and rotation angles are composed of multiplication and addition operations with sub1, sub2, sub3, sub4, as above, and have a similar structure, differing only in the noise values used. The final error value $Pt_{E}$ used is equal to the product of the error values for distance, angle, and rotation angle.

Since the associated likelihood function part takes the output of the previous step, predicted measurements, and returns a simplified form of the final output, the two parts are bundled and parallelized. The ratio of the two parts in the algorithm is about 21.56%. By bundling the two parts, we not only increase the simplicity of the design but also save data transfer time between PS and PL. To parallelize this part, we use Xilinx Vitis HLS to synthesize the hardware IP. Algorithm 3 shows pseudo-code for the predicted measurements part and the associated likelihood function part. This part consists of a single iteration, where the estimation and the calculation of the quality of the particles through the likelihood function are repeated as many times as the number of particles.
**Algorithm 3:** Predicted Measurements and Associated Likelihood FunctionInputParticle’s position information:$\;Pt_{p1},\;Pt_{p2},\;Pt_{p3}$ Distance, angle, rotation angle measurement: $mea_{D},\;mea_{\theta },\;mea_{\psi }$
OutputParticle Quality $Pt_{E}$
1.**for** *i* =0: the number of particles2.   calculate estimation of distance **Equation (2)**3.   calculate estimation of angle **Equation (3)**4.   calculate estimation of rotation angle **Equation (4)**5.   calculate error of distance **Equation (5)**6.   $\mathrm{calculate}\;sub_{1},\;sub_{2},\;sub_{3},\;sub_{4}$ **Equations (6)–(9)**7.   calculate error of angle **Equation (10)**8.   $\mathrm{calculate}\;sub_{1},\;sub_{2},\;sub_{3},\;sub_{4}$ **Equations (6)–(9)**9.   calculate error of rotation angle **Equation (10)**10.   calculate error of particle **Equation (11)**11.**end**

**Figure 6:**
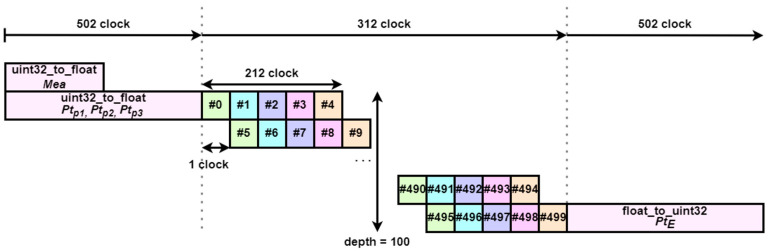
Parallelized predicted measurements and associated likelihood function block diagram.

The design of the parallel computation hardware IP to accelerate the predicted measurements and the associated likelihood function part is completed as follows. First, Algorithm 4 shows the pseudo-code for parallelizing this part. In this hardware IP, the number of particles entering the input is set to 500, and data type casting is performed as in Section 4.3. to transfer data. The computational part of this section is pipelined and performed in parallel. When pipelining, if the time for the operation to be performed is too short, the pipelining depth will decrease at the same time, and the efficiency of pipelining will decrease. Therefore, in this section, instead of parallelizing the operation for one particle, we parallelize the sequential processing of five particles. As a result, the operations for 5 particles are overlapped every 1 clock. The result of this pipelining can be seen in Figure 6. The total time it takes to perform the operations on the five particles is 212 clocks, so the pipelining depth is set to 212 to allow 212 operations to be performed simultaneously. Although 212 operations can be performed simultaneously, the loop is repeated a total of 100 times, so all operations are performed simultaneously.
**Algorithm 4:** Parallelized Predicted Measurements and Associated Likelihood FunctionInputParticle’s position information uint32 array:$\;Pt_{p1},\;Pt_{p2},\;Pt_{p3}$ Distance, angle, rotation angle measurement uint32 array: mea
OutputParticle Quality uint32 array$\;Pt_{E}$
1.**do in sequential: for** *x* = 0: the number of particles2.   uint32_to_float_datatype_casting($Pt_{p1})$3.   uint32_to_float_datatype_casting($Pt_{p2})$4.   uint32_to_float_datatype_casting($Pt_{p3})$
5.**end**6.**do in sequential: for** *x* = 0:27.   uint32_to_float_datatype_casting(mea)
8.**end**9.**do in parallel: for** *i* = 0: the number of particles/510.   **do in sequential: for *j* = 0:4**
11.      calculate estimation of distance **Equation (2)**12.      calculate estimation of angle **Equation (3)**13.      calculate estimation of rotation angle **Equation (4)**14.      calculate error of distance **Equation (5)**15.      $\mathrm{calculate}\;sub_{1},\;sub_{2},\;sub_{3},\;sub_{4}$ **Equations (6)–(9)**16.      calculate error of angle **Equation (10)**17.      $\mathrm{calculate}\;sub_{1},\;sub_{2},\;sub_{3},\;sub_{4}$ **Equations (6)–(9)**18.calculate error of rotation angle **Equation (10)**19.calculate error of particle **Equation (11)**20.   **end**
21.**end**22.**do in sequential: for** *x* = 0: the number of particles23.   float_to_uint32_datatype_casting($Pt_{E})$
24.**end**

### 4.5. Parallelization Method #3: Update Particles

The update particles part, which updates the particle’s information, takes up about 30.00% of the total algorithm and is the second most time-consuming part of the algorithm. This process is performed for the preset number of epochs in each iteration of the PSO, and the operation is performed for each particle. This part of the algorithm is time-consuming and the computation time increases with the number of particles and the number of epochs, so it needs to be parallelized. The process of updating a particle’s information is defined as follows.

(24) $$Pt_{a+1}=kai\times \left(\left(c\times eps\times \left(OP_{g}-Pt_{p}\right)\right)+\left(c\times eps\times \left(OP_{l}-Pt_{p}\right)\right)\right)-\left(1-kai\right)\times Pt_{V}$$

(25) $$Pt_{v+1}=Pt_{v}+Pt_{a+1}$$

(26) $$Pt_{p+1}=Pt_{p}+Pt_{v+1}$$

where $Pt_{a}$, $Pt_{v}$, and $Pt_{p}$ are the current acceleration, velocity, and position of a particle, and $Pt_{a+1}$, $Pt_{v+1}$, and $Pt_{p+1}$ are the acceleration, velocity, and position of the particle in the next epoch, respectively. In addition, $OP_{g}$ is the global optimal point for all particles and $OP_{l}$ is the local optimal point. Using the equations above, it is possible to calculate the information the particle will have in the next cycle from the current particle’s information. The design of the parallel computation hardware IP to accelerate the update particles part is completed as follows. First, Algorithm 5 shows the pseudo-code for the update particles part before parallelization. The algorithm proceeds as long as the current epoch is not the last epoch and iterates over each particle, updating the values in each dimension of the particle.
**Algorithm 5:** Update ParticlesInputParticle’s information in current cycle:$\;Pt_{p},\;Pt_{v}$ Global Optimal Point: $OP_{g}$ Local Optimal Point: $OP_{l}$ Current epoch number $N_{c}$
OutputParticle’s information in next cycle:$\;Pt_{p+1},\;Pt_{v+1},\;Pt_{a+1}$
1.**If** $N_{c}$ **< *max cycle number***2.   **for** *i* = 0: the number of particles3.     **for** *j* = 0: the number of particle’s dimension
        **for** *k* = 0:44.           $\mathrm{Calculate}\;Pt_{a+1}$ **Equation (12)**5.           $\mathrm{Calculate}\;Pt_{v+1}$ **Equation (13)**6.           $\mathrm{Calculate}\;Pt_{p+1}$ **Equation (14)**
         **end**
7.      **end**
8.   **end**
9.**end**

**Figure 7:**
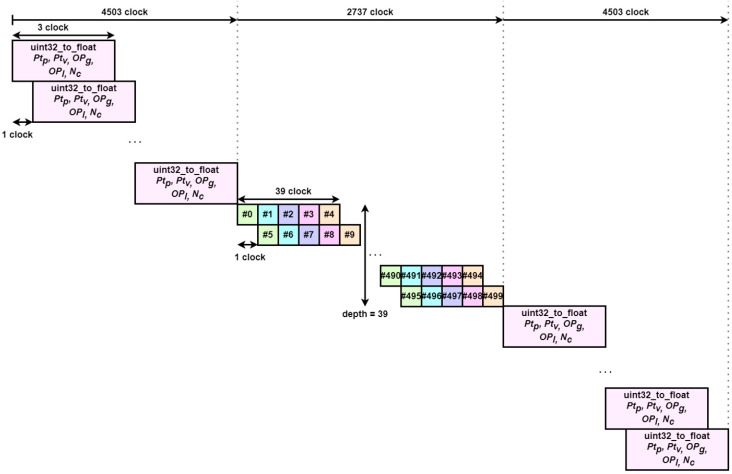
Parallelized update particles block diagram.

Next, Algorithm 6 shows pseudo-code for the update particles part after parallelization. The number of particles input to the hardware IP is set to 500 as in the previous method, and we also perform data type casting to transfer the data. The data type casting part of this part takes 3 clocks per iteration, so it can be performed in parallel. We parallelize the update operations for the five particles by pipelining them as we did for the main computation Methods #3–4. The result of this pipelining can be seen in Figure 7. The computation for the five particles takes a total of 39 clocks, and the pipeline depth is set to 39 to perform 39 operations in parallel. This part of the algorithm is performed 100 times for each of the 5 particles and the dimensionality of the particles is 9, so 900 iterations.
**Algorithm 6:** Parallelized Update ParticlesInputParticle information for the current cycle uint32 array:$\;Pt_{p},\;Pt_{v}$ Global Optimal Point uint32 array: $OP_{g}$ Local Optimal Point uint32 array: $OP_{l}$ Current epoch number: $N_{c}$
OutputParticle’s information for the next cycle uint32 array:$\;Pt_{p+1},\;Pt_{v+1},\;Pt_{a+1}$
1.**do in parallel: for** *x* = 0: the number of particles2.   uint32_to_float_datatype_casting ($Pt_{p})$
3.   uint32_to_float_datatype_casting ($Pt_{v})$
4.   uint32_to_float_datatype_casting ($OP_{l})$
5.   **if** *x* < particle’s dimension:6.      uint32_to_float_datatype_casting ($OP_{g})$
7.   **end**
8.**end**9.**do in parallel**: **for** *i* = 0: the number of particles/510.   **do in parallel: for** *j* = 0: the number of particle’s dimension11.      **do in sequential: for** *k* = 0:412.         $\mathrm{Calculate}\;Pt_{a+1}$ **Equation (12)**13.         $\mathrm{Calculate}\;Pt_{v+1}$ **Equation (13)**14.         $\mathrm{Calculate}\;Pt_{p+1}$ **Equation (14)**15.    **end**
16.**end**17.**end**18.**do in parallel: for** *x* = 0: the number of particles19.   float_to_uint32_datatype_casting ($Pt_{p+1})$
20.float_to_uint32_datatype_casting ($Pt_{v+1})$
21.float_to_uint32_datatype_casting $\left(Pt_{a+1}\right)$
22.**end**

### 4.6. Hardware Platform Design

**Figure 8:**
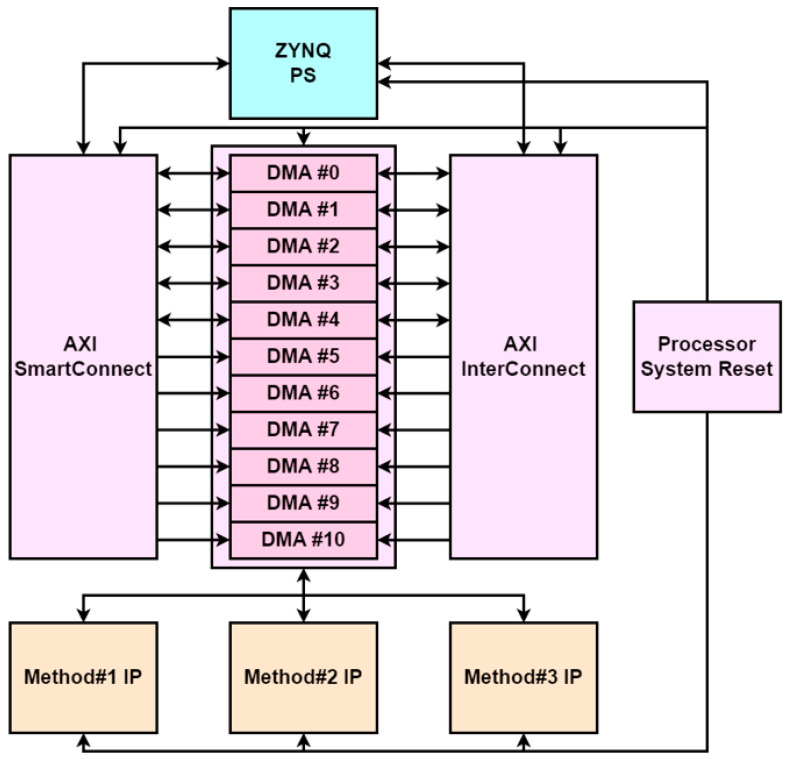
Hardware platform design block diagram.

Next, design a platform to connect the PS and PL of the zc706 using the hardware IP designed in Method #1, Method #2, and Method #3. The design of the platform is completed in Vivado 2022.1. Figure 8 shows the block diagram of the designed hardware. The design of the platform was accomplished as follows. First, the three custom hardware IPs communicate via the AXI-Stream protocol, which utilizes AXI Direct Memory Access (DMA). DMA is a hardware IP that provides AXI memory mapping and also provides high-bandwidth direct memory access between peripherals, and we used the IP provided by Vivado out of the box. We used 11 DMAs to account for the number of I/O ports in the three custom IPs. We also used the AXI Smartconnect, Interconnect IP provided by Vivado for mapping between master and slave devices.

## 5. Results

### 5.1. Hardware Platform Design Results

The hardware platform design was carried out through the methods proposed in Section 4. First, the heterogeneous device co-design was performed on the AMD Zynq 7000 SoC ZC706 Evaluation Kit (xc7z045ffg900-2) with dual ARM Cortex-A9 core processors. Table 2 shows the hardware usage of the ballistic trajectory target tracking application with PSO and the total amount of available hardware resources on the xc7z045ffg900-2. The maximum hardware resources available to the xc7z045ffg900-2 are 218,600 LUTs, 70,400 LUTRAMs, 437,200 FFs, 900 DSPs, and 545 BRAMs, and the application utilizes 40.51%, 7.45%, 21.72%, 41.67%, and 18.35% of the hardware resources.

Next, the power consumption of the created hardware platform is shown in Table 3. The static power consumption of the device is about 0.232 W, which is very low. Furthermore, when the internal resources are maximally utilized, the device consumes 2.876 W of power, of which the processing system has the highest share. In conclusion, the proposed hardware platform consumes a maximum of 3.108 W and a minimum of 0.232 W on the device.

### 5.2. Simulation Results

In this paper, four parts of the PSO algorithm of the ballistic target tracking algorithm are accelerated using an FPGA to achieve realtime performance. Using the above methods, the parallelized ballistic target tracking algorithm is tested in a simulation environment. To simulate a real missile in a simulation environment, the dynamic model in Equations (1) and (2) is used, as described earlier. The aerodynamic drag and weight of the debris are referenced to [32], the sampling interval is set to ∆t = 0.01 s, and the total simulation time is 3 s. The deviations $n_{d},\;n_{\theta },\;n_{\psi }$ of the radar receiver noise model are 0.1 m, 0.1 deg, and 0.1 deg, respectively, and the glint noise $n_{G\theta },\;n_{G\psi }$ follows a Gaussian distribution as follows.

(27) $$p=\left(1-\alpha \right)p_{G_{1}}+\alpha p_{G_{2}}$$

where α is the glint probability, $p_{G_{1}}$ is a Gaussian model with $p_{G_{1}}\sim N\left(0,0.1^{2}\right)$, and $p_{G_{2}}$ is a Gaussian model with $p_{G_{2}}\sim N\left(0,1^{2}\right)$. The tracking motion model is a Singer model according to Equation (5), and the measurement model is obtained using Equation (12). The position of the radar is assumed to be fixed on the ground, and the ballistic target is assumed to move at high speed considering gravity and aerodynamic drag. The parameters of the PSO algorithm were set as follows: c, which determines the speed when moving from the local optimum to the global optimum, was set to 2.05; kai, which indicates that the particles maintain their current speed, was set to 0.729843788; and eps, which determines how far the particles will spread out when the algorithm starts, was set to a random number less than or equal to 1. In addition, epoch, the number of times the particle moves, and the number of particles were set differently for each experiment to check the difference in experimental results.

**Figure 9:**
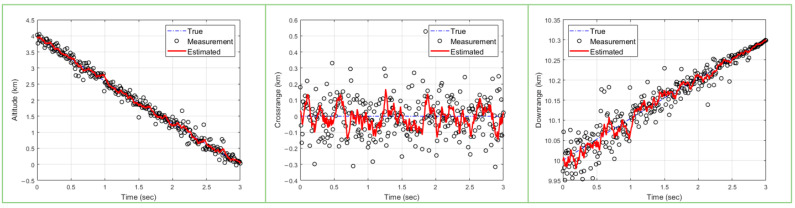
Ballistic target tracking results with 500 particles and 15 epochs (blue dashed line: true/circle: measured/red solid line: tracking results).

**Figure 10:**
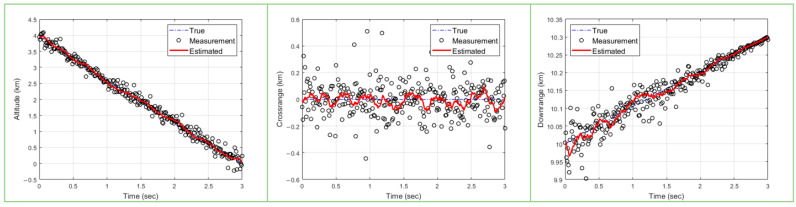
Ballistic target tracking results with 3000 particles and 10 epochs (blue dashed line: true/circle: measured/red solid line: tracking results).

The results of trajectory and state estimation are shown in Figure 9 and Figure 10. In Figure 9 and Figure 10, the left plot shows the estimated altitude range compared to the true model, the middle plot shows the crossrange compared to the true model, and the right plot shows the target downrange compared to the true model. The performance of the particle swarm optimization algorithm depends on the number of particles and the number of epochs. First, Figure 9 shows the results of an experiment with 500 particles and 15 epochs. Next, Figure 10 shows the results of an experiment with 3000 particles and 10 epochs per particle for comparison. It can be seen that, when the number of particles and the number of epochs are small, the convergence is not very good at the beginning and the error bound bounces a great deal, as shown in Figure 9. On the other hand, in Figure 10, we can see that, when the number of particles and the number of epochs are sufficient, the optimal value is found well, unlike in Figure 9. First, in the altitude direction, we observe an initial bouncing of the error bounds, but we can see that the optimal value is found and maintained through iteration since the simulation has been running for 1.5 s. Also, in the crossrange direction, the error bound bounces in the later part compared to the earlier part, but it does not deviate much. Finally, in the downrange direction, we can see that the optimal value is well found. In all three directions, we can see that the optimal value is found and maintained through the iteration process, so we can see that the target is being tracked normally.

Compared to other metaheuristic algorithms, the PSO algorithm still shows strong performance [33,34,35,36]. In this paper, the results of the PSO-based ballistic target tracking were compared with the results of the ballistic target tracking with Monte Carlo Optimization, which is a well-known metaheuristics algorithm. The comparison results of the errors in downrange, crossrange, altitude, and overall mean squared errors (MSE) according to the number of particles or samples are summarized in Table 4. The errors with the PSO decrease according to the number of particles more significantly than the errors with the MCO, which means that the PSO is more appropriate than the MCO in the context of accuracy because more particles are required for more accurate estimation. Therefore, this paper chose the PSO as an algorithm for ballistic target tracking.

### 5.3. Results of Algorithm Acceleration with FPGA

The parallelization and acceleration results in this paper were performed on an AMD Zynq 7000 SoC ZC706 Evaluation Kit (xc7z045ffg900-2) with an FPGA and Dual ARM Cortex-A9 core processor. The board has 218,600 LUTs, 5244 LUTRAMs, 437,200 FFs, 545 BRAMs, and 900 DSPs in hardware resources. In this section, we compare the non-parallelized particle swarm optimization technique using PS only and the parallelized results using PS and PL together on xc7z045ffg900-2.

First, the parallelization results for each part are shown in Table 5, Table 6, Table 7, Table 8 and Table 9. Table 5 shows the computation time according to the number of particles in the Method #1 random value to particles part with 10 epochs. For this part, we present the experimental results according to the number of particles because it is independent of the number of epochs and is only affected by the number of particles. In this part, you can see that the computation time increases linearly as the number of particles increases. Similarly, the parallelized results also show a linear increase, but the increase is much smaller due to parallelization. As a result, the acceleration gain for this part is about 7.45×.

Table 6 and Table 7 show the experimental results according to the number of particles and the number of epochs in the Method #2 predicted measurements and associated likelihood functions part. First, Table 6 shows the experimental results according to the number of particles when the number of epochs is 10. The results show an acceleration of about 5.94 to 7.11 times. Table 7 shows the computation time results according to the number of particles when the number of particles is 1500. The results show an acceleration of about 5.3 to 7.2 times.

Table 8 and Table 9 show the results of the experiment according to the number of particles and the number epochs in the Method #3 update particles part. First, Table 8 shows the experimental results according to the number of particles when the number of epochs is 10. From Table 8, we can see that the acceleration is about 1.40 to 1.48 times. Table 9 shows the computation time as a function of the number of epochs when the number of particles is 1500. It shows a time accelerated by about 1.34 to 1.68 times.

Table 10 shows a comparison of the execution time of the PSO algorithm as a function of the number of particles when the number of epochs is 10. When comparing the performance of parallel acceleration based on the execution time of PSO, the acceleration is about 3.01 to 3.24 times. Table 11 shows the comparison of the execution time of the PSO algorithm according to the number of epochs when the number of particles is 1500. The results show a speedup of about 2.34 to 3.89 times.

Table 12 shows the execution time comparison for the entire target tracking algorithm according to the number of particles when the number of epochs is 10. When comparing the performance of the parallel acceleration based on the total algorithm execution time, we can see that the acceleration is about 2.63× to 2.83×. Table 13 shows the execution time comparison for the entire target tracking algorithm as a function of the number of epochs when the number of particles is 1500. When comparing the performance of parallel acceleration based on the total algorithm execution time, we can see a speedup of about 2.30× to 3.43×.

### 5.4. Analysis and Discussion

First, in Table 2, which shows the hardware platform design results, the proposed heterogeneous co-designed application uses about 7.45% to 41.67% of the total resources of the FPGA part, xc7z045ffg900-2. In the acceleration part of the ballistic trajectory target tracking algorithm, the size of the output data is small compared to the amount of data computation, and it contains a large number of addition, multiplication, and trigonometric function operations. Therefore, it can be seen that the computational resources, such as LUT and DSP, are consumed more than the memory resources, such as LUTRAM, FF, and BRAM. Next, check Table 3 to see the power consumption of the designed hardware. The PS, which corresponds to the CPU, is about 1.568 W, which accounts for about 50.4% of the total power consumption, and the remaining 1.540 W, or 49.6%, is used to operate the FPGA. From this, it is clear that the overall power consumption remains the same or lower at 3.108 W, and the amount of power used by the FPGA is very low.

Next, Figure 9 shows that the PSO-based ballistic trajectory target tracking algorithm fails to track the target when the number of particles and epochs are insufficient. As mentioned before, due to the sampling-based nature of the algorithm, the performance of the algorithm decreases rapidly if a sufficient number of iterations are not provided. However, in Figure 10, it is clear that, when enough iterations are performed, the tracking is successful in all directions.

Next, looking at Table 5, Table 6 and Table 7, the three parts of the algorithm that account for 64.87% of the total algorithm, excluding the update particles, show a significant acceleration of 5.30 to 7.49 times. However, if looking at Table 8 and Table 9, the update particles part, which accounts for about 30% of the total algorithm, is accelerated by about 1.34 to 1.68 times. The reason for the lower acceleration of the update particles part compared to the other parts is that the input data are huge compared to the previous parts, and therefore it takes a long time for the CPU to flatten the two-dimensional array into a one-dimensional array in order to move the data between PS and PL using the AXI protocol. While Method #1 takes the position information of a single initialized particle and Method #2 takes the position information of all particles as input, the update particles part requires the position information, velocity information, acceleration information, global optimum point, and local optimum point of all particles as input, so it takes a great deal of time to flatten from the data structure used by the CPU to a one-dimensional array.

As a result, checking Table 12 and Table 13, applying the parallelized particle swarm optimization method results in an acceleration of about 2.34 to 3.89 times over the PSO algorithm alone and about 2.30 to 3.43 times over the ballistic target tracking algorithm as a whole. The reasons why the acceleration increases when the number of particles increases and decreases when the number of epochs increases are analyzed as follows: first, the amount of non-parallelized parts of ballistic trajectory target tracking increases when the number of epochs increases, and the amount of acceleration decreases because the share of update particles parts that do not have a large effect increases. However, even with all of these limitations, it can be concluded that, for ballistic target tracking, designing heterogeneous devices with FPGA increases realtime performance because it takes less computation time than using only CPU to track the target’s state.

## 6. Conclusions

In designing a guided missile for intercepting ballistic missiles, an accurate target estimation algorithm is essential to track and intercept the target missile. PSO algorithms can be a solution to this problem because they can overcome the challenges of the nonlinear and non-Gaussian nature of real-world noise and can handle a wide range of error distributions. In practice, the performance of PSO-based ballistic trajectory target tracking algorithms has been verified and showed that they can accurately estimate altitude, crossrange, and downrange. However, due to the nature of sampling-based optimization algorithms, the computational time burden increases significantly as the number of particles increases or the number of particle movements increases, so a large number of iterations greatly reduces the realtime performance of the algorithm. To solve these problems, this paper overcomes the limitations through the mutual optimal design of heterogeneous devices such as CPU and FPGA. The computation time of the ballistic trajectory target tracking algorithm was analyzed on the CPU, and the part that takes a long time due to the iteration structure was parallelized on the FPGA. Four parts of the ballistic trajectory target tracking algorithm were selected as targets, and they were accelerated by 1.34 to 7.45 times. As a result, the algorithm was accelerated by 2.30 to 3.43 times, and the computation time was significantly reduced, improving realtime performance. We also verified the results in terms of power consumption. Using the low power consuming characteristics of the FPGA, the result was about 3.109 W, which is significantly lower than the typical power consumption of a GPU, which is a typical device with parallel processing characteristics. However, the limitation of this study is that it focused on parallelization through pipelining of tasks. This parallelization method proved to be effective in accelerating the algorithm, but there is still potential for other parallelization methods, such as vectorization and systolic. Our future work will be to experimentally apply various parallelization methods to the parallelization of the target estimation algorithm and to apply better methods to increase the acceleration of the algorithm.

## Figures and Tables

**Table 1:** Works related to the acceleration of PSO.

Related Works	Parallelization	Parallelization Part	Missile Application
[15,16,17]	X	-	X
[18,19,20]	X	-	O
[21]	GPU	Propagate particle swarm Compute objective error Update “best” Update positions	X
[22]	GPU	Calculation of error	X
[23]	GPU	Calculation of the cost Function	X
[24]	FPGA	Initialization Evaluation Position update	X
[25]	FPGA	Particle updating	X
[26]	FPGA	Particle updating Finding best particle	X
[27]	FPGA	Calculation of Pearson’s correlation coefficient	X
Ours	FPGA	Random value to particles Predicted measurements Associated likelihood function Particle update	O

**Table 2:** Application’s hardware resource usage.

Resource	Utilization	Available	Utilization (%)
LUT	88,558	218,600	40.51
LUTRAM	5244	70,400	7.45
FF	94,978	437,200	21.72
BRAM	100	545	18.35
DSP	375	900	41.67

**Table 3:** Power consumption of the designed hardware platform.

	Static	Dynamic					
	Device	Clocks	Signals	Logic	BRAM	DSP	PS
Power Consumption(W)	0.232	0.194	0.507	0.455	0.038	0.114	1.568
Ratio (%)	7.5	6.2	16.3	14.6	1.2	3.7	50.4

**Table 4:** Comparison of the errors with MCO and PSO for ballistic target tracking.

Particle (Samples)	Errors with MCO (km)				Errors with PSO (km)			
	Downrange	Crossrange	Altitude	MSE	Downrange	Crossrange	Altitude	MSE
500	0.0133	0.0319	0.0627	0.072	0.0117	0.0524	0.0555	0.077
1000	0.0151	0.0220	0.0638	0.069	0.0158	0.0257	0.0698	0.076
1500	0.0115	0.0319	0.0499	0.060	0.0080	0.0376	0.0420	0.057
2000	0.0127	0.0246	0.0493	0.057	0.0079	0.0283	0.0358	0.046
2500	0.0102	0.0299	0.0417	0.052	0.0085	0.0254	0.0363	0.045

**Table 5:** Result of computation time according to the number of particles in the random value to particles part.

Number of Particles	PS Only (ms)	PS + PL (ms)	Acceleration (×)
500	11,361.02	1524.74	7.45
1000	22,695.18	3044.62	7.45
1500	34,327.68	4602.71	7.46
2000	45,456.47	6071.05	7.49
2500	57,199.55	7675.52	7.45
3000	68,115.51	9105.85	7.48

**Table 6:** Result of computation time according to the number of particles in the predicted measurements and associated likelihood functions part.

Number of Particles	PS Only (ms)	PS + PL (ms)	Acceleration (×)
500	5326.16	748.85	7.11
1000	11,142.67	1653.90	6.74
1500	16,948.53	2595.53	6.53
2000	22,470.24	34,876.89	6.44
2500	28,299.83	4621.18	6.12
3000	33,907.71	5710.24	5.94

**Table 7:** Result of computation time according to the number of epochs in the predicted measurements and associated likelihood functions part.

Number of Epochs	PS Only (ms)	PS + PL (ms)	Acceleration (×)
5	8046.33	1110.77	7.24
10	16,948.53	2595.52	6.53
15	26,041.55	4768.62	5.46
20	34,477.19	6496.31	5.31
25	43,515.75	8208.57	5.30

**Table 8:** Result of computation time according to the number of particles in the update particles part.

Number of Particles	PS Only (ms)	PS + PL (ms)	Acceleration (×)
500	7520.93	5356.54	1.40
1000	15,439.34	10,694.70	1.44
1500	24,290.23	16,393.26	1.48
2000	31,390.19	21,727.67	1.44
2500	40,636.12	27,443.28	1.48
3000	47,189.01	32,950.91	1.43

**Table 9:** Result of computation time according to the number epochs in the update particles part.

Number of Epochs	PS Only (ms)	PS + PL (ms)	Acceleration (×)
5	9603.01	7146.623	1.34
10	24,290.23	16,392.51	1.48
15	41,529.85	25,573.33	1.62
20	62,120.61	39,063.76	1.59
25	81,150.53	48,362.21	1.68

**Table 10:** When the number of epochs is 10, the particle swarm optimization algorithm execution time according to the number of particles.

Number of Particles	PS Only (ms)	PS + PL (ms)	Acceleration (×)
500	24,796.47	8256.29	3.25
1000	50,601.06	16,767.23	3.16
1500	77,585.86	25,727.49	3.12
2000	102,039.91	34,199.77	3.08
2500	129,611.31	43,473.34	3.06
3000	153,392.16	52,223.67	3.01

**Table 11:** When the number of particles is 1500, the particle swarm optimization algorithm execution time according to the number of epochs.

Number of Epochs	PS Only (ms)	PS + PL (ms)	Acceleration (×)
5	52,384.83	13,459.42	3.89
10	77,585.86	25,724.92	3.02
15	105,879.64	39,467.58	2.68
20	137,987.21	57,831.46	2.39
25	169,369.51	72,241.48	2.34

**Table 12:** When the number of epochs is 10, the entire algorithm execution time according to the number of particles.

Number of Particles	PS Only (ms)	PS + PL (ms)	Acceleration (×)
500	26,790.02	10,168.72	2.63
1000	52,984.94	19,031.35	2.78
1500	80,338.84	28,373.23	2.83
2000	105,170.13	37,193.46	2.82
2500	133,116.34	46,854.30	2.84
3000	157,289.37	55,978.17	2.81

**Table 13:** When the number of particles is 1500, the entire algorithm execution time according to the number of epochs.

Number of Epochs	PS Only (ms)	PS + PL (ms)	Acceleration (×)
5	55,014.56	16,033.68	3.43
10	80,338.84	28,361.65	2.83
15	108,783.71	42,171.31	2.58
20	140,860.32	60,564.57	2.33
25	172,240.61	74,963.45	2.30
